# Alterations in Gastric Mucosal Expression of Calcitonin Gene-Related Peptides, Vanilloid Receptors, and Heme Oxygenase-1 Mediate Gastroprotective Action of Carbon Monoxide against Ethanol-Induced Gastric Mucosal Lesions

**DOI:** 10.3390/ijms19102960

**Published:** 2018-09-28

**Authors:** Katarzyna Magierowska, Dagmara Wojcik, Anna Chmura, Dominik Bakalarz, Mateusz Wierdak, Slawomir Kwiecien, Zbigniew Sliwowski, Tomasz Brzozowski, Marcin Magierowski

**Affiliations:** Department of Physiology, Faculty of Medicine, Jagiellonian University Medical College, 31-531 Cracow, Poland; k.jasnos@interia.pl (K.M.); dagmara1.wojcik@uj.edu.pl (D.W.); anna.1.chmura@uj.edu.pl (A.C.); dominik.bakalarz@uj.edu.pl (D.B.); mateusz.wierdak@uj.edu.pl (M.W.); skwiecien@cm-uj.krakow.pl (S.K.); zbigniew.sliwowski@uj.edu.pl (Z.S.); mpbrzozo@cyf-kr.edu.pl (T.B.)

**Keywords:** carbon monoxide, gastric mucosa, transient receptor potential vanilloid receptor type 1, calcitonin gene-related peptide

## Abstract

Carbon monoxide (CO) has been reported to contribute to the maintenance of gastric mucosal integrity, gastroprotection, and ulcer healing. However, involvement of transient receptor potential vanilloid receptor type 1 (TRPV1) located on afferent sensory fibers endings and sensory neuropeptide calcitonin gene-related peptide (CGRP) in CO-mediated gastroprotection against ethanol-induced gastric damage has not been explored. Male Wistar rats with and without denervation of afferent sensory neurons induced by capsaicin (total dose 125 mg/kg within 3 days) were pretreated with vehicle, CO donor tricarbonyldichlororuthenium (II) dimer (CORM-2, 5 mg/kg i.g.), administered alone or with CGRP-α (10 μg/kg i.p.) or TRPV1 antagonist capsazepine (5 mg/kg i.g.), followed 30 min later by intragastric (i.g.) administration of 75% ethanol. The area of gastric damage and gastric blood flow (GBF) were assessed planimetrically and by laser flowmetry, respectively. Microscopic evaluation of ethanol-induced gastric lesions was performed after haematoxylin/eosin (H&E) or alcian blue/periodic acid-Schiff/alcian blue (AB/PAS) staining. Gastric mucosal mRNA fold change for heme oxygenase (HMOX)-1, HMOX-2, CGRP-α, CGRP-β, inducible nitric oxide synthase (iNOS), endothelial (e)NOS, neuronal (n)NOS, cyclooxygenase (COX)-1, COX-2, and protein expression for HMOX-1 and TRPV1 was determined by real-time PCR or Western blot, respectively. Pretreatment with CORM-2 combined or not with CGRP reduced ethanol-induced gastric lesions and elevated GBF. Capsaicin-denervation or co-treatment with capsazepine or CGRP and CORM-2 in capsaicin-denervated animals failed to affect these beneficial effects of CO donor. In rats with intact sensory nerves, CORM-2 increased gastric mRNA level for HMOX-1 and CGRP-α. In capsaicin-denervated rats, CORM-2 increased eNOS mRNA fold change and TRPV1 protein expression while capsaicin denervation itself decreased HMOX-1 protein expression and eNOS mRNA level. We conclude that CO prevents gastric mucosa from ethanol-induced lesions due to activation of TRPV1/CGRP-α system and accompanying increase in gastric microcirculation but independently on afferent sensory nerve activity despite the stimulation of TRPV1 protein and CGRP-α mRNA expression.

## 1. Introduction

Gastric mucosal integrity is maintained by the activity of various physiological factors such as endogenous prostaglandins, nitric oxide (NO), hydrogen sulfide (H_2_S), and mechanisms such as mucus producing and bicarbonates secreting epithelial cells, gastric microcirculation, and expression and activity of scavengers of reactive oxygen species (ROS) [1,2,3]. However, necrotizing agents i.e., ethyl alcohol, drugs such as non-steroid anti-inflammatory drugs (NSAIDs) (e.g., aspirin) or stress are considered as the major factors responsible for the disruption of gastric mucosal barrier causing a fall in the gastric blood flow (GBF) and an impairment of protective lines of mucosal defense system [4,5]. Gastric microcirculation is regulated by the activity of afferent sensory neurons, known to release the vasoactive mediator calcitonin gene-related peptide (CGRP) through the activation of transient receptor potential vanilloid receptor type 1 (TRPV1) at the site of inflammation [6,7,8].

Besides NO and H_2_S, carbon monoxide (CO) has also recently been reported to play an essential role in the maintenance of gastric mucosal integrity due to its anti-inflammatory and vasoactive properties involved in the regulation of GBF [9,10,11]. Recently our group reported that gaseous mediators NO, H_2_S, and CO, exhibited gastroprotective activity but in contrast to NO and H_2_S, the CO-induced protection was found to be independent on the activity of afferent sensory neurons [8,11]. However, the contribution of TRPV1 and CGRP to CO-mediated gastroprotection against ethanol-induced gastric lesions has not been fully investigated and needs further evaluation.

Therefore, in this study we aimed to evaluate if CO released from its pharmacological donor, tricarbonyldichlororuthenium (II) dimer (CORM-2) can prevent the rat gastric mucosa against ethanol-induced gastric damage in TRPV1/CGRP-dependent manner. We also determined the alterations in gastric mucosal mRNA, protein expression, or both, for (a) enzymes involved in endogenous CO production, heme oxygenase (HMOX)-1, and HMOX-2 [8], (b) key enzymes of endogenous prostaglandin biosynthesis, cyclooxygenase (COX)-1, and COX-2 [8], and (c) crucial enzymes responsible for production of endogenous NO, constitutive endothelial NOS (eNOS) and neuronal NOS (nNOS), and inducible NO synthase (iNOS) [8,11] in CORM-2 pretreated rats with intact or capsaicin-denervated sensory nerves receiving 75% ethanol.

## 2. Results

**Figure 1:**
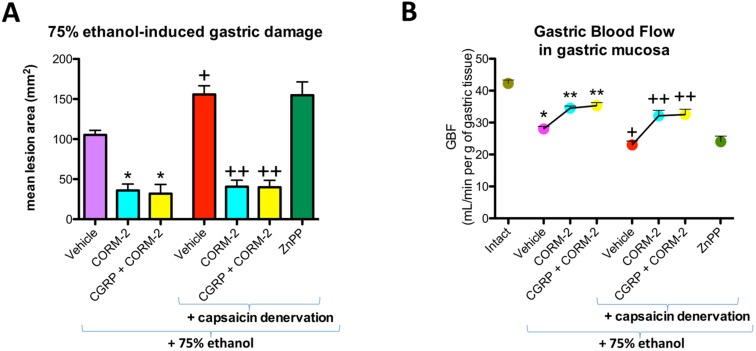
Mean area of 75% ethanol-induced gastric damage (**A**) and gastric blood flow (GBF) (**B**) in rats with or without afferent sensory neurons ablation (capsaicin denervation) and pretreated with vehicle, CORM-2 (5 mg/kg i.g.), ZnPP (5 mg/kg i.g.) alone or in combination with CGRP-α (10 μg/kg i.p.). Intact refers to healthy gastric mucosa, not exposed to 75% ethanol. Results are mean ± SEM of 4–5 rats per each experimental group. (**A**) Significant change as compared with the respective values in vehicle-control group without capsaicin denervation was indicated by asterisk or cross (*p* < 0.05); double crosses indicate significant changes compared to the values obtained in the vehicle-pretreated group with capsaicin denervation (*p* < 0.05); (**B**) Significant change as compared with the respective values in intact gastric mucosa was indicated by asterisk (*p* < 0.05); double asterisks indicate significant changes compared to the values obtained in the vehicle-pretreated group without capsaicin denervation (*p* < 0.05); cross indicates a significant change as compared with the respective vehicle-pretreated and capsaicin-denervated animals (*p* < 0.05); double crosses indicate significant changes compared to the values obtained in the vehicle-pretreated group with capsaicin denervation (*p* < 0.05).

Figure 1 shows that pretreatment with CORM-2 (5 mg/kg i.g.) administered alone or in combination with CGRP-α (10 μg/kg i.p.) significantly decreased the mean area of ethanol-induced gastric damage and significantly increased GBF as compared with vehicle-pretreated control group of rats with and without capsaicin denervation of afferent sensory neurons (*p* < 0.05) (Figure 1A,B). This observation partly confirmed our previously published evidence that CORM-2 prevented ethanol-induced gastric damage and that this effect was accompanied by an increase in GBF [12]. ZnPP administered to rats with capsaicin denervation did not significantly affect ethanol-induced damage area or GBF (Figure 1A,B). Capsaicin denervation itself significantly increased the area of gastric damage induced by ethanol and significantly decreased GBF as compared with vehicle-control group without capsaicin-sensory nerves ablation (*p* < 0.05) (Figure 1A,B).

**Figure 2:**
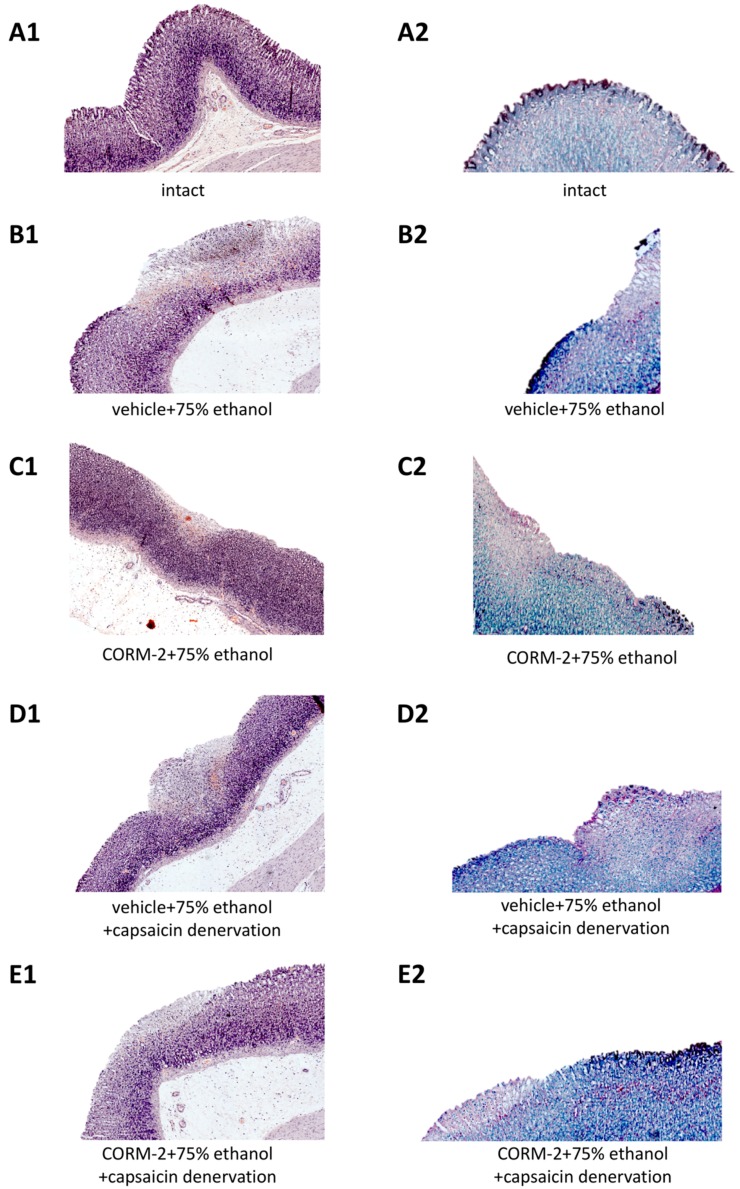
Microscopic appearances of H&E (**A1**–**E1**) or AB/PAS (**A2**–**E2**) stained intact gastric mucosa and gastric mucosa of rats with or without capsaicin denervation, exposed to 75% ethanol and pretreated with vehicle or CORM-2 (5 mg/kg i.g.).

The microscopic appearance of intact gastric mucosa and gastric injury in rats administered with 75% ethanol is shown in Figure 2(A1–E2). Figure 2(A1) shows representative histology photomicrographs of intact gastric mucosa stained with H&E and AB/PAS (Figure 2(A1,A2), respectively) and vehicle-control gastric mucosa treated with 75% ethanol (Figure 2(B1,B2)). In rats pretreated with vehicle with or without capsaicin denervation, administration of 75% ethanol resulted in necrotic erosions deeply penetrating into gastric mucosa with significant leukocytes infiltration into submucosal layer with accompanying extensive desquamation of epithelium surface (Figure 2(B1,D1) vs. Figure 2(A1)). Mucus layer on the surface of the gastric mucosa was completely reduced and glycoprotein content was intense in submucosal part exposed to 75% ethanol as observed in AB/PAS staining and compared with histological appearance of intact healthy gastric mucosa (Figure 2(B2,D2) vs. Figure 2(A2)). Increased glycoproteins production was observed in healthy gastric mucosa surrounding necrotic ethanol-induced damage (Figure 2(B2,D2)).

In gastric mucosa of rats with or without capsaicin denervation and pretreated with CORM-2, ethanol-induced epithelial damage was limited only to superficial layer and less white blood cells infiltration was observed as compared with vehicle-control samples (Figure 2(C1,E1) vs. Figure 2(B1,D1), respectively). However, disrupted by ethanol continuity of mucosal lining and mucus layer was not completely preserved in rats pretreated with CORM-2 with and without sensory nerves ablation as compared to vehicle-control (Figure 2(C2,E2) vs. Figure 2(B2,D2), respectively).

**Figure 3:**
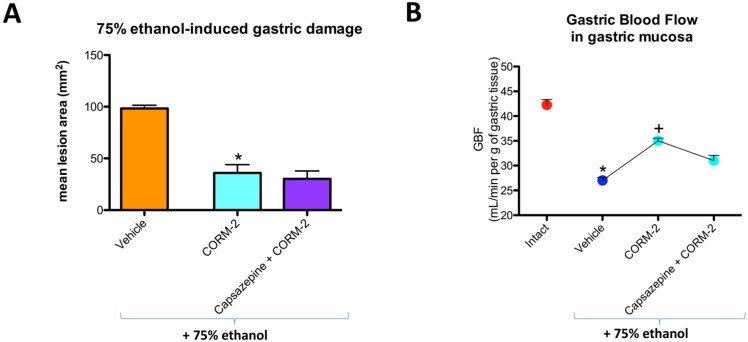
Mean area of 75% ethanol-induced gastric damage (**A**) and GBF (**B**) in rats pretreated with vehicle or CORM-2 (5 mg/kg i.g.) alone or in combination with capsazepine (5 mg/kg i.g.). Intact refers to healthy gastric mucosa, not exposed to 75% ethanol. Results are mean ± SEM of 4–5 rats per each experimental group. (**A**) Significant change as compared with the respective values in vehicle-control group was indicated by asterisk (*p* < 0.05); (**B**) Significant change as compared with the respective values in intact gastric mucosa was indicated by asterisk (*p* < 0.05); cross indicates significant change compared to the values obtained in the vehicle pretreated group (*p* < 0.05).

Capsazepine (5 mg/kg i.g.) failed to affect CORM-2-induced decrease in the area of ethanol-induced gastric lesions and the increase in GBF as compared with the respective values in rats pretreated with CORM-2 alone (Figure 3A,B).

**Figure 4:**
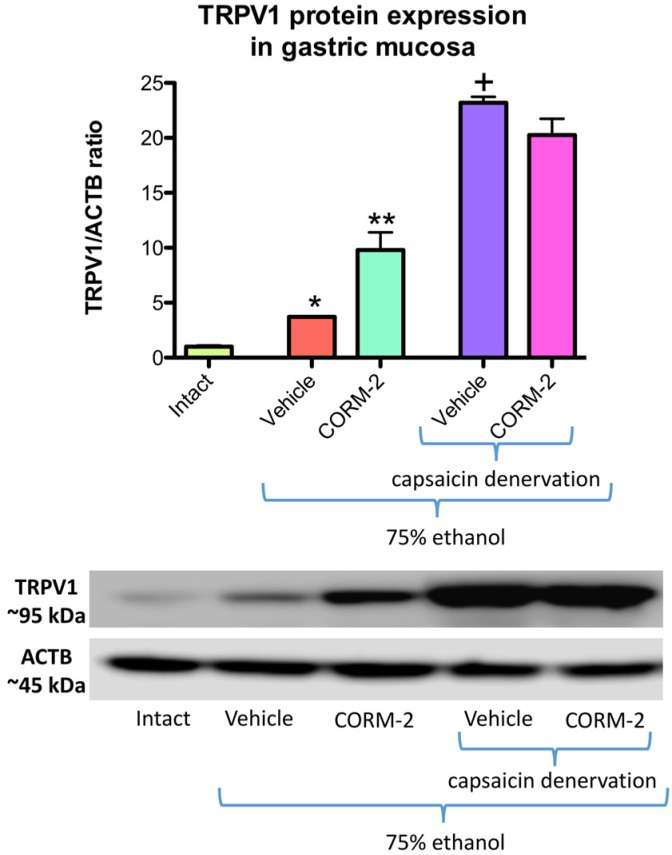
Protein expression of TRPV1 in gastric mucosa of rats pretreated with vehicle or CORM-2 (5 mg/kg i.g.) and administered i.g. with 75% ethanol with or without capsaicin denervation. Intact refers to healthy gastric mucosa. Results are expressed as protein expressions of TRPV1 normalized to β-actin. Results are mean ± SEM of 4–5 rats per group. Asterisk indicates a significant change as compared with values obtained in intact gastric mucosa (*p* < 0.05). Double asterisks or cross indicates significant change as compared with respective values obtained in vehicle-pretreated group without capsaicin denervation (*p* < 0.05).

Figure 4 shows that protein expression for TRPV1 is significantly increased in gastric mucosa administered with 75% ethanol as compared with intact rats (*p* < 0.05). Pretreatment with CORM-2 (5 mg/kg i.g.) significantly upregulated TRPV1 protein expression as compared with vehicle-control group (*p* < 0.05) (Figure 4). After administration of 75% ethanol to vehicle pretreated rats with capsaicin denervation, the expression of gastric mucosal TRPV1 protein was significantly increased as compared with gastric mucosa administered with 75% ethanol in rats with intact sensory nerves (*p* < 0.05) (Figure 4). Pretreatment with CORM-2 failed to affect TRPV1 protein expression as compared with vehicle-control group of rats with capsaicin denervation and administered with 75% ethanol (Figure 4).

**Figure 5:**
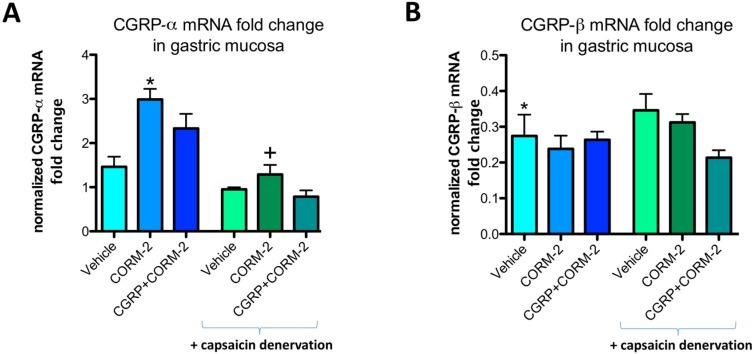
CGRP-α (**A**) and CGRP-β (**B**) mRNA fold change in gastric mucosa of rats with or without capsaicin denervation and pretreated with vehicle, CORM-2 (5 mg/kg i.g.) alone or in combination with exogenous CGRP-α (10 μg/kg i.p.) and administered i.g. with 75% ethanol. Intact refers to healthy gastric mucosa. Results are expressed as mRNA fold change of CGRP-α or CGRP-β normalized to succinate dehydrogenase complex, subunit A (SDHA) and β-actin. Results are mean ± SEM. of 4–5 rats per group. (**A**) Asterisk indicates a significant change as compared with vehicle-control group without capsaicin denervation (*p* < 0.05); cross indicates a significant change as compared with the group treated with CORM-2 without capsaicin-denervation. (**B**) Asterisk indicates significant change as compared with values obtained in intact gastric mucosa (*p* < 0.05).

Figure 5A shows that CGRP-α mRNA fold change in gastric mucosa of vehicle-pretreated rats administered with 75% ethanol was not significantly affected as compared with respective values in intact gastric mucosa. Pretreatment with CORM-2 significantly increased the mRNA fold change for CGRP-α as compared with respective vehicle-control group in rats without capsaicin denervation (*p* < 0.05) but not in those with capsaicin-induced sensory denervation (Figure 5A). In gastric mucosa of rats with capsaicin denervation, CGRP-α mRNA fold change was not significantly affected as compared with vehicle-control group with intact sensory nerves (Figure 5A). Figure 5B shows that CGRP-β mRNA fold change in gastric mucosa pretreated with vehicle and administered with 75% ethanol was significantly decreased as compared with respective values in intact gastric mucosa and this fall in fold change of CGRP-β mRNA remained without significant change in capsaicin denervated animals with or without CORM-2 combined or not with CGRP.

**Figure 6:**
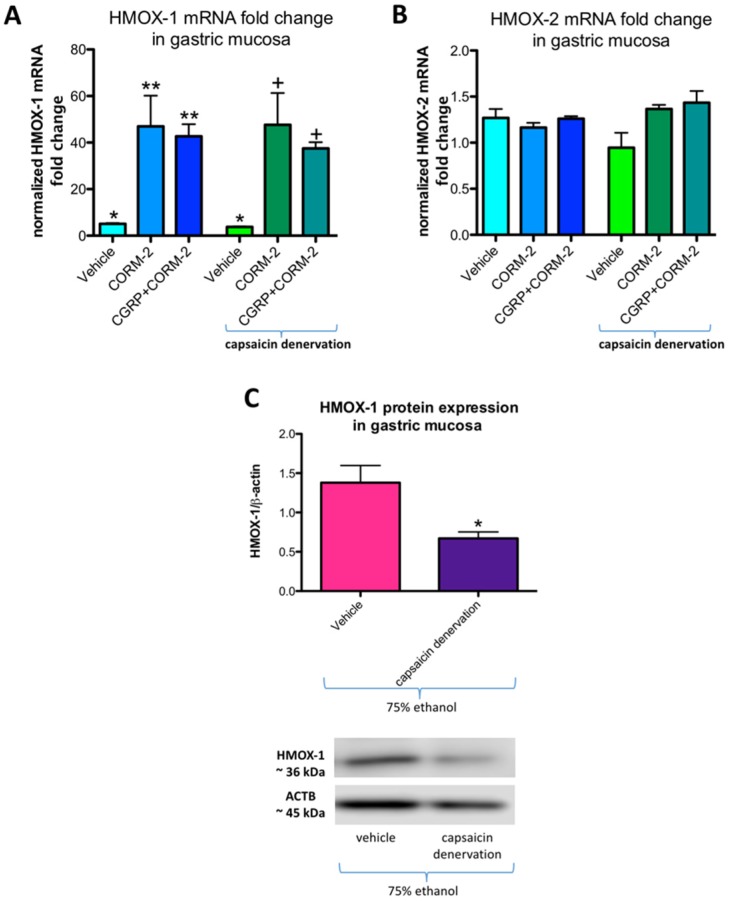
HMOX-1 (**A**) and HMOX-2 (**B**) mRNA fold change in gastric mucosa of rats with or without capsaicin denervation and pretreated with vehicle or CORM-2 (5 mg/kg i.g.) alone or with CGRP-α (10 μg/kg i.p.) followed by i.g. administration of 75% ethanol. Panel **C**: gastric mucosal HMOX-1 protein expression in rats administered with 75% ethanol with and without capsaicin denervation. Results are expressed as mRNA fold change of HMOX-1 and HMOX-2 normalized to succinate dehydrogenase complex, subunit A (SDHA) and β-actin. Results are mean ± SEM of 4–5 rats per each experimental group. (**A**) Asterisk indicates a significant change as compared with intact rats (*p* < 0.05); double asterisks indicate significant difference as compared with vehicle pretreated group without capsaicin denervation (*p* < 0.05); cross indicates significant change as compared with vehicle pretreated group with capsaicin denervation (*p* < 0.05). (**C**) Asterisk indicates significant change as compared with gastric mucosa administered with 75% ethanol of rats without capsaicin denervation (*p* < 0.05).

Figure 6A,B show mRNA fold change for HMOX-1 and HMOX-2, respectively, in rats pretreated with vehicle or CORM-2 (5 mg/kg i.g.) with and without capsaicin denervation and followed by i.g. application of 75% ethanol. In gastric mucosa of vehicle-control rats administered with ethanol with and without capsaicin denervation, HMOX-1 mRNA fold change was significantly increased as compared with intact rats (*p* < 0.05) (Figure 6A). Pretreatment with CORM-2 alone or combined with CGRP-α a further upregulation of this fold change was observed as compared to vehicle-control groups with and without capsaicin denervation (*p* < 0.05) (Figure 6A). These observations partly confirm our previously published results that 75% ethanol upregulated gastric mucosal HMOX-1 mRNA fold change and pretreatment with CORM-2 enhanced this effect [12]. In gastric mucosa of rats with capsaicin denervation who received ethanol, HMOX-1 mRNA fold change was not significantly affected as compared with vehicle-control group with intact sensory nerves (Figure 6A). Gastric mucosal HMOX-2 mRNA fold change was not significantly affected by 75% ethanol neither in rats without nor with capsaicin denervation (Figure 6B). Figure 6C shows that HMOX-1 protein expression was significantly downregulated in gastric mucosa exposed to 75% ethanol in vehicle-control rats with capsaicin denervation as compared with animals without sensory denervation administered with ethanol (*p* < 0.05).

**Figure 7:**
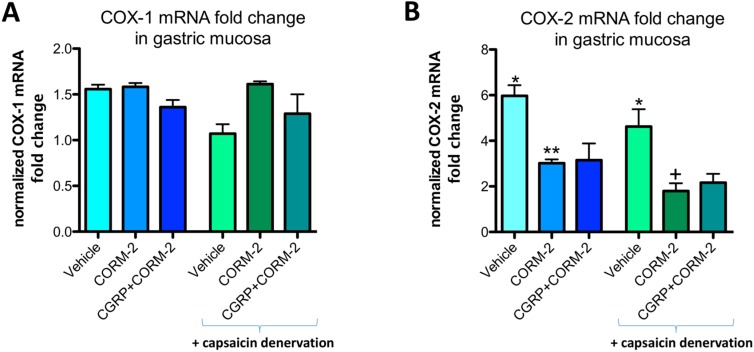
COX-1 (**A**) and COX-2 (**B**) mRNA fold change in gastric mucosa of rats pretreated with vehicle or CORM-2 (5 mg/kg i.g.) alone or with CGRP-α (10 μg/kg i.p.) followed by i.g. administration of 75% ethanol with or without capsaicin denervation. Results are expressed as mRNA fold change of COX-1 and COX-2 normalized to succinate dehydrogenase complex, subunit A (SDHA) and β-actin. Results are mean ± SEM of 4–5 rats per each experimental group. Asterisk indicates significant change as compared with intact rats (*p* < 0.05); double asterisks indicate significant difference as compared with vehicle-pretreated group without capsaicin denervation (*p* < 0.05); cross indicates significant change as compared with vehicle-pretreated group with capsaicin denervation (*p* < 0.05).

Gastric mucosal COX-1 mRNA fold change in vehicle-control rats was not significantly affected by the application of 75% ethanol neither in rats without nor with capsaicin denervation (Figure 7A). In gastric mucosa of rats with and without capsaicin denervation and with ethanol-induced gastric damage, COX-2 mRNA fold change was significantly increased as compared with intact rats (*p* < 0.05) (Figure 7B). Pretreatment with CORM-2 administered alone or combined with CGRP-α significantly decreased this COX-2 mRNA fold change as compared to respective vehicle-control groups with and without capsaicin denervation (*p* < 0.05) (Figure 7B).

**Figure 8:**
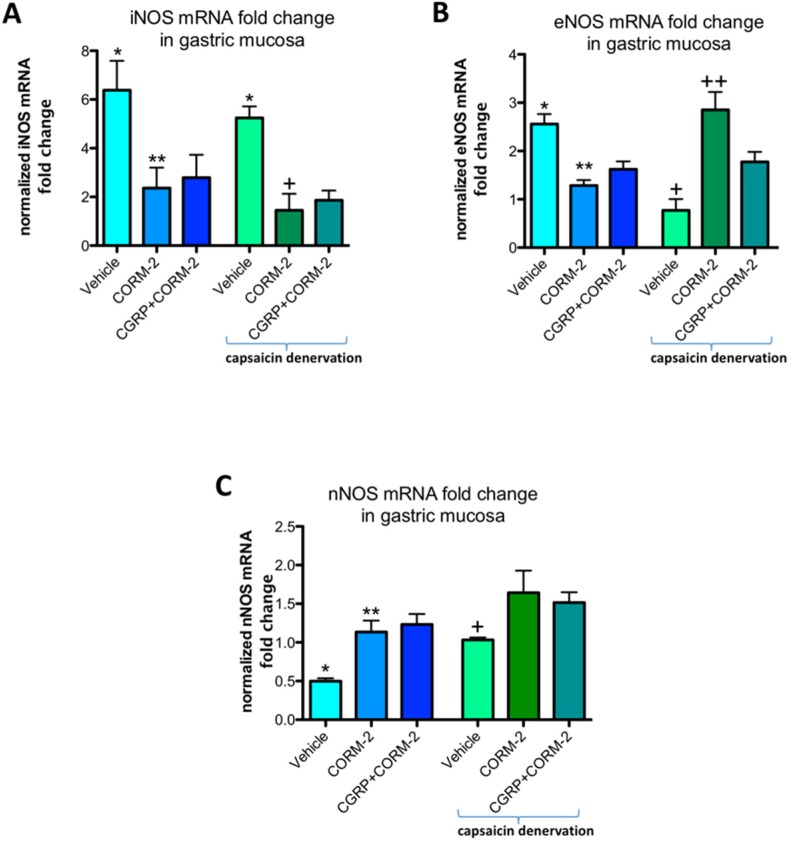
iNOS (**A**), eNOS (**B**), and nNOS (**C**) mRNA fold change in gastric mucosa of rats with or without capsaicin denervation and pretreated with vehicle or CORM-2 (5 mg/kg i.g.) alone or with CGRP-α (10 μg/kg i.p.) followed by i.g. administration of 75% ethanol. Results are expressed as mRNA fold change of iNOS, eNOS, and nNOS normalized to succinate dehydrogenase complex, subunit A (SDHA) and β-actin. Results are mean ± SEM of 4–5 rats per each experimental group. (**A**) Asterisk indicates significant change as compared with intact rats (*p* < 0.05); double asterisks indicate significant difference as compared with vehicle pretreated group without capsaicin denervation (*p* < 0.05); cross indicates significant change as compared with vehicle pretreated group with capsaicin denervation (*p* < 0.05); (**B**) Asterisk indicates significant change as compared with intact rats (*p* < 0.05); double asterisks indicate significant difference as compared with vehicle pretreated group without capsaicin denervation (*p* < 0.05); cross indicates significant change as compared with vehicle pretreated group without capsaicin denervation (*p* < 0.05); double crosses indicate significant difference as compared with vehicle pretreated group with capsaicin denervation (*p* < 0.05); (**C**) Asterisk indicates significant change as compared with intact rats (*p* < 0.05); double asterisks or cross indicates significant difference as compared with vehicle pretreated group without capsaicin denervation (*p* < 0.05).

In gastric mucosa of rats with and without capsaicin denervation and with ethanol-induced gastric damage, iNOS mRNA fold change was significantly increased as compared with intact rats (*p* < 0.05) (Figure 8A). Pretreatment with CORM-2 administered alone or combined with CGRP-α downregulated this expression as compared to respective vehicle-control groups with and without capsaicin denervation (*p* < 0.05) (Figure 8A). Gastric mucosal eNOS mRNA fold change was upregulated by 75% ethanol in rats without capsaicin denervation (*p* < 0.05) (Figure 8B). Pretreatment with CORM-2 administered alone or combined with CGRP-α significantly decreased this expression as compared to vehicle-control group without capsaicin denervation (*p* < 0.05) (Figure 8B). In capsaicin denervated animals, 75% ethanol downregulated gastric mucosal eNOS mRNA fold change as compared with vehicle-pretreated control group administered with 75% ethanol without sensory denervation (*p* < 0.05) (Figure 8B). Pretreatment with CORM-2 administered alone or combined with CGRP-α significantly increased eNOS mRNA fold change as compared to vehicle-pretreated rats with capsaicin denervation exposed to 75% ethanol (*p* < 0.05) (Figure 8B). Pretreatment with CORM-2 administered alone or combined with CGRP-α significantly increased nNOS mRNA fold change as compared to vehicle-pretreated rats without capsaicin denervation exposed to 75% ethanol (*p* < 0.05) (Figure 8C).

## 3. Discussion

Afferent sensory C neuronal fibers play an important role in regulation of gastric microcirculation and therefore in the maintenance of gastric mucosal integrity by the mechanism involving release of vasoactive CGRP due to activation of TRPV1 [6,7,11,13]. Interestingly, endogenous gaseous mediators NO, H_2_S, and CO have been shown to increase GBF and prevent gastric mucosa from the formation of acute gastric mucosal lesions induced by intragastric application of aspirin, ethanol, strong acid and bases, or exposure of experimental animals to cold stress [8,11,12]. Moreover, the cross-talk between NO or H_2_S and afferent sensory nerves activity is considered to be essential element for the functionality of gastric mucosal barrier [11]. Previously, we have demonstrated that locally increased bioavailability of CO prevented gastric mucosa against ethanol-induced necrotic damage and that this effect was accompanied by the enhancement in GBF [12]. This observation has been confirmed also in other studies showing that CO prevents gastric mucosa against necrotic damage by its interaction with 5’ adenosine monophosphate-activated protein kinase [14]. However, the interaction of neuromediator released from afferent C fiber endings with gaseous molecule CO in the pathogenesis of ethanol-induced gastric damage still remains unexplored.

To our best knowledge we provided for the first time the experimental evidence that mechanism of gastroprotective effect of CO released from its pharmacological donor, CORM-2 against necrotic ethanol-induced gastric damage involves the upregulation of TRPV1 protein expression in gastric mucosa. First of all, we have observed that application of 75% ethanol dramatically decreased GBF and that this effect coincides with an increase in gastric mucosal protein expression of TRPV1. In contrast, the gastroprotective effect of CORM-2 was accompanied by elevation of GBF and enhancement of this TRPV1 expression suggesting that CO exerts the gastroprotective activity due to its vasoactive properties possibly mediated by the CO-induced stimulation of vanilloid receptors located at afferent sensory nerves. Further support for this notion that the afferent sensory nerves can be involved in CORM-2-induced gastroprotection against ethanol damage comes out from our observation that CORM-2 increased mRNA fold change for CGRP-α, the neuropeptide released from afferent nerve endings which is known to act through TRPV1 to exert its gastroprotective and vasorelaxatory activities including an increase in GBF. Interestingly, besides the originally discovered localization at intrinsic enteric neurons and extrinsic sensory neurons, TRPV1 has also been identified in epithelial and endocrine cells [15]. Moreover, the co-localization of immunoreactive CGRP released from afferent neurons with TRPV1 receptors has been documented throughout the gastrointestinal tract including stomach [16]. Besides the enhancement in gastric microcirculation, the beneficial effects of CGRP on gastric mucosa include inhibition of gastric acid secretion, prevention of cellular apoptosis and the attenuation of oxidative injury [17]. Furthermore, the agonists of TRPV1 were reported to exert considerable potential for gastric mucosal protection [17].

However, the concominant treatment of CORM-2 with exogenous CGRP-α failed to considerably enhance the genuine gastroprotective effects of CO donor when administered alone against ethanol-induced damage and the accompanying gastric hyperemia observed in CORM-2 pretreated animals. This is in keeping with previously published observation on CO and CGRP relationship demonstrating that the pharmacological inhibition of HMOX-1 which decreased production of endogenous CO also downregulated CGRP-α mRNA expression in dorsal root ganglia [18]. Moreover, it has been shown in other systems that the increased CGRP concentration and expression HMOX-1 protein were correlated in patients with Barrter/Gitelman syndromes [19]. We assume that ethanol-induced damage and immunity changes associated with this inflammatory signaling could activate a self-defense response of gastric mucosa as reflected by the increased TRPV1 protein expression. Furthermore, CO released from CORM-2 applied prior ethanol enhanced this beneficial mucosal response possibly by a physiological feedback mechanism resulting in acceleration of this effect of CORM-2 on CGRP-α fold change in a consequence leading to vasodilatation, elevation of GBF and finally attenuating the severity of ethanol-induced gastric damage.

On the other hand, capsaicin denervation of afferent sensory neurons or pharmacological inhibition of TRPV1 by capsazepine failed to reduce gastroprotective and vasodilatory effect of CO released from CORM-2 in gastric mucosa compromised by ethanol. This is in pair with previously published data showing that capsaicin denervation failed to affect gastroprotective effect of CO against aspirin- or stress-induced gastric lesions [8,11]. Moreover, the CO-mediated decrease of gastric mucosal mRNA fold change of proinflammatory COX-2 and iNOS was not affected in ethanol-treated rats with capsaicin-induced sensory denervation. Interestingly, we observed that the gastric mucosal protein or mRNA expression of HMOX-1 and eNOS, key enzymes responsible for the production of endogenous CO or NO production, respectively, were both decreased in gastric mucosa of rats with capsaicin-induced denervation of afferent sensory neurons and reversed CORM-2-induced upregulation of CGRP-α mRNA expression. Furthermore, pharmacological inhibition of HMOX-1 by ZnPP did not affected ethanol-induced gastric damage or GBF. Similarly, Peng et al. reported that high dose of capsaicin abrogated HMOX-1 expression in dorsal root ganglia and therefore enhanced monophosphoryl lipid A (MLA)-induced delayed cardioprotection [20]. On the other hand, activation of TRPV1 by low doses of capsaicin increased mRNA expression for HMOX-1 and ameliorated cisplatin-induced nephrotoxicity [21]. Additionally, TRPV1 agonist, glyceryl nonivamide upregulated HMOX-1 expression and inhibited lipopolysaccharide-activated microglia-mediated neuronal death [22]. We assume, that in our ethanol model, the decreased content of endogenous CO and NO as a result of capsaicin denervation weakened gastric mucosal barrier and therefore potentiated ethanol-induced gastric damage formation. Because CORM-2 increased gastric mucosal eNOS mRNA fold change in rats with sensory neurons ablation and nNOS mRNA fold change in rats with intact sensory fibers, we conclude that the CO-mediated gastroprotection and vasorelaxation under these conditions with depletion of sensory neuropeptides, mainly CGRP, could involve increased bioavailability of NO despite the suppression of sensory nerve activity by capsaicin. Indeed, in our previous studies we have observed that CO gastroprotection against ethanol-induced gastric damage is reduced when NOS activity was pharmacologically inhibited [12]. We assume that capsaicin-induced aggravation of ethanol-induced gastric damage is due to decreased HMOX-1 activity. We conclude that it is also possible that exogenous CO directly acts on gastric microcirculation and increases blood flow by its vasodilatory effect, rather than mediated by afferent nerve activation via TRPV1 and CGRP since CORM-2 increased eNOS expression under capsaicin denervation. Therefore, exogenous CO may increase blood flow directly or via nitric oxide (NO) production from eNOS and nNOS.

## 4. Materials and Methods

### 4.1. Experimental Design: Animals, Chemicals, and Drugs Treatment Regime

Fifty male Wistar rats 8–9 weeks old and weighting 220–300 g were fasted for 24 h with free access to drinking water before all chemicals and drugs application. All rats were fed with standard food and water under the environment with temperature 20 ± 2 °C, humidity 50–60% and 12 h light/dark cycle. The study was approved by the Institutional Animal Care and Use Committee of Jagiellonian University Medical College in Cracow (Approval no 68/2014, permission date: 21 May 2014; Approval no 137/2012, permission date: 19 September 2012) and run in accordance with the statements of the Helsinki Declaration regarding handling of experimental animals. Experiments were run with implications for replacement, refinement or reduction (the 3Rs) principle. Animal studies are reported in compliance with the ARRIVE guidelines.

Two major series of rats A and B were employed in our study. In series A, rats with intact sensory nerves were used whereas in rats of series B capsaicin was administered for three days in a dose of 25, 50 and 50 mg/kg (total dose: 125 mg/kg s.c.) two weeks before start of the study, to induce functional ablation of afferent sensory nerves as described previously [11]. High dose of capsaicin has been demonstrated previously to induce complete afferent sensory neurons ablation [8].

In the day of experiment, animals of both series A and B were randomly divided into appropriate experimental groups, 4–5 animals each and were pretreated either with: (1) vehicle (DMSO/saline (1:4); 1 mL/rat), (2) CORM-2 administered intragastrically (i.g.) in a gastroprotective dose of 5 mg/kg, selected in our previous study [12] applied alone or combined with CGRP-α (10 μg/kg i.p.) [8], (3) zinc protoporphyrin IX (ZnPP, 5 mg/kg i.g.), an inhibitor of HMOX [12], all applied 30 min before i.g. application of 75% ethanol. In group 3, rats were pretreated with CORM-2 administered with or without the combination with TRPV1 antagonist, capsazepine (5 mg/kg i.g.), before 75% ethanol instillation [23].

To induce necrotic gastric damage in control experiments, the 75% ethanol was administered i.g. to vehicle-pretreated control animals in the volume of 1.5 mL using orogastric tube, 30 min after chemicals and drugs application, as reported by our group previously [12]. This model based on 75% ethanol is known to induce necrotic gastric mucosal damage and to downregulate GBF [12].

All tested compounds were of analytical grade and were purchased from Sigma-Aldrich (Schnelldorf, Germany).

### 4.2. Measurement of GBF and Macroscopical and Microscopical Assessment of Ethanol-Induced Gastric Damage

One hour after 75% ethanol administration, animals were anesthetized with pentobarbital (60 mg/kg i.p.), their abdomens were opened and GBF was determined in exposed stomachs by laser flowmetry as described previously [11]. Briefly, the GBF was measured in oxyntic part of the gastric mucosa not involving mucosal lesions. Average values of three measurements were determined and expressed in mL/min per 100 g of gastric tissue. The area of ethanol-induced gastric damage in each rat stomach was determined planimetrically and expressed in mm^2^ [11,12].

For histology, the gastric tissue sections were excised and fixed in 10% buffered formalin, pH 7.4. Samples were dehydrated by passing them through a series of alcohols with incremental concentrations, equilibrated in xylene for 10–15 min and embedded in paraffin; paraffin blocks were cut into about 4 μm sections using a microtome. The prepared specimens were stained with haematoxylin/eosin (H&E) or alcian blue/periodic acid-Schiff/alcian blue (AB/PAS) and evaluated using a light microscope (AxioVert A1, Carl Zeiss, Oberkochen, Germany) [24]. Digital documentation of histological slides was obtained using above mentioned microsope equipped with automatic scanning table and ZEN Pro 2.3 software (Carl Zeiss, Oberkochen, Germany) to collect multiple photographs of each histological sample and to stitch them into one picture; to obtain better quality of each picture, the background was subtracted and unified as white.

Gastric mucosal biopsies (about 500 mg) were collected on ice, snap-frozen in liquid nitrogen and stored at −80 °C for further analysis [25].

### 4.3. Determination of mRNA Level Fold Change for Heme Oxygenase Enzymes HMOX-1, HMOX-2, CGRP-α, CGRP-β, iNOS, eNOS, nNOS, COX-1, COX-2 in Gastric Mucosa by Real Time Polymerase Chain Reaction (PCR)

Gastric mucosal mRNA expression for HMOX-1, HMOX-2, CGRP-α, CGRP-β, iNOS, eNOS, nNOS, COX-1, and COX-2 was determined by semi-quantitative real time PCR, as described previously [24].

Total RNA was isolated using commercially available kit with spin-columns (GeneMATRIX Universal RNA Purification Kit, EURx, Gdansk, Poland) according to manufacturer protocol. Reversed trascription to cDNA was performed using PrimeScript™ RT Master Mix (Perfect Real Time) (Takara Bio Inc., Kyoto, Japan). RNA concentration was measured using Qubit 4 Fluorometer (Thermo Fisher Scientific, Waltham, MA, USA). RT was normalized for each reaction regarding total RNA concentration to obtain the same value (1 µg) for each sample. Results obtained for RNA samples isolated from healthy (intact) gastric mucosa and transcribed to cDNA were further used as reference control during calculations.

Fold change for HMOX-1, HMOX-2, CGRP-α, iNOS, COX-1, COX-2, β-actin mRNA levels was determined using specific primers [11,24,25,26]. Succinate dehydrogenase complex, subunit A (SDHA) as reference gene was determined using 5’-TCCTTCCCACTGTGCATTACAA-3’ forward and 5’-CGTACAGACCAGGCACAATCTG-3’ reverse primers. CGRP-β was determined using 5’-GAGGTGTGGTGAAGGACAAC-3’ forward and 5’-CAACTTTATGTAACCTTCTTCCTGG-3’ reverse primers. eNOS was determined using 5’-CCTGGCAGCCCTAAGACCTA-3’ forward and 5’-GACGCTGGTTGCCATAGTGA-3’ reverse primers. nNOS was determined using 5’-ACCATCGTTGACCACCACTC-3’ forward and 5’-GACATGGGAGGCACAATCCA-3’ reverse primers. SDHA and β-actin were used as reference genes.

The real time PCR was conducted using thermal cycler Quant Studio 3 (Thermo Fisher Scientific, Waltham, MA, USA) and SYBR Green I dye including kit (SG qPCR Master Mix (2×), EURx, Gdansk, Poland). The same amount of cDNA per each well was used to maintain the same PCR reaction efficiency in all analyzed samples. After reaction, melting curve for each sample, its technical replicates and for appropriate negative control were analyzed to exclude the data derived from potentially unintended products. Results were analyzed using the −ΔΔ*C*_t_ method [12,27].

### 4.4. Analysis of Protein Expression of HMOX-1, TRPV1 and β-actin in Gastric Mucosa by Western Blot

Protein expressions for HMOX-1, TRPV1, and β-actin in gastric mucosa were determined using western blot as described in our previous studies [24,25]. Briefly, rabbit monoclonal anti-HMOX-1 (ab68477, Abcam, Cambridge, UK) in dilution of 1:2000, mouse monoclonal anti-TRPV1 (ab203103, Abcam) in dilution of 1:1000, mouse monoclonal anti-β-actin (3700S, Cell Signaling Technology, Danvers, MA, USA) in dilution of 1:1000 were used as primary antibodies. Protein expression was visualized using horseradish peroxidase-linked secondary anti-rabbit IgG antibody (7074, Cell Signaling Technology) or anti-mouse IgG antibody (7076, Cell Signaling Technology) in dilution of 1:2000 where appropriate. All primary and secondary antibodies were diluted in 5% non-fat milk.

Chemiluminescence was developed using WesternSure^®^ ECL Substrate (LI-COR, Lincoln, NE, USA) or WesternBright Quantum (Advansta, Menlo Park, CA, USA) and was measured using C-DiGit^®^ Blot Scanner (LI-COR). The intensity of bands was determined and analysed using Image Studio 4.0 software (LI-COR). The expression of each protein of interest was determined using 5 samples per experimental group and obtained values were normalized to the expression of β-actin as loading control [24].

HMOX-1 is known to exert time-dependent discrepancies between mRNA and protein levels in damaged gastric mucosa [12]. While TRPV1 is a crucial receptor for afferent neurons activity and the main aim of this study [6,7,11]. Therefore, we investigated those two targets on protein level. Other abovementioned targets were measured on mRNA level because majority of them were previously considered as the markers of gastric damage induced by ethanol or other noxious factors and we aimed to show how CORM-2 and capsaicin-denervation affects their mRNA profile [11,12,24,25].

### 4.5. Statistical Analysis

Results were analyzed using GraphPad Prism 5.0 software (GraphPad Software, La Jolla, CA, USA). Results are presented as mean ± SEM. Statistical analysis was conducted using Student’s t-test or ANOVA with Dunnett’s Multiple Comparison post hoc test if more than two experimental groups were compared. The group size for each experimental group was of *n* = 4 or 5. *p* < 0.05 was considered as statistically significant.

## 5. Conclusions

Taken together, we conclude that CO released from CORM-2 attenuated ethanol-induced gastric mucosal lesions in rats with intact sensory nerves and in those with functional ablation of afferent sensory nerves induced by capsaicin. This militates against the possible mediation of this CO-induced gastroprotection by the stimulation of sensory nerve activity by this gaseous molecule despite the fact that an increase in expression of TRPV1 protein and CGRP-α mRNA expression in gastric mucosa of CO-pretreated animals has been observed. It is possible that besides NO as manifested by an increase in eNOS expression and anti-inflammatory activity of CO as documented by the fall in COX-2 and iNOS expression in rats with and without sensory denervation, the TRPV1/CGRP-α system can be involved in gastric hyperemia associated with this protection induced by CO released from its donor, CORM-2. Exogenous CO may increase blood flow directly or via nitric oxide (NO) production from eNOS and nNOS. However, the mechanism of CO-dependent gastroprotection and vasorelaxation observed in the stomach injured by ethanol seems to be more complex and the role of afferent sensory fibers activity in this beneficial effect of CO in gastroprotection against noxious agents including ethanol warrants further investigation.
